# A Case of Docetaxel-Induced Rhabdomyolysis

**DOI:** 10.7759/cureus.9380

**Published:** 2020-07-25

**Authors:** Nishanth Thalambedu, Muhammad Umair Atiq, Sheel Patel

**Affiliations:** 1 Internal Medicine, Abington Hospital - Jefferson Health, Abington, USA; 2 Hematology and Oncology, Thomas Jefferson University, Philadelphia, USA

**Keywords:** docetaxel, chemotherapy, breast cancer, rhabdomyolysis

## Abstract

Docetaxel is an antimicrotubule agent with activity in a variety of cancers. Its toxicity profile includes myelosuppression, fluid retention/edema, and peripheral neuropathy. It is also associated with myalgias but the frequency and extent of this toxicity are not well described. Here, we present a case of a 48-year-old female with breast cancer who developed bilateral proximal leg pain and inability to walk two weeks after the third cycle of docetaxel and cyclophosphamide. Initial workup showed elevated creatinine kinase (CK) levels which trended up to 9000 U/L suggesting rhabdomyolysis. She was treated with IV isotonic fluids without renal complications. Her CK was 1800 U/L at discharge and normalized two weeks post-discharge. To our knowledge, this is one of the few reports of docetaxel-induced rhabdomyolysis. The purpose of this report is to alert physicians of this rare but morbid complication.

## Introduction

Rhabdomyolysis is a condition due to muscle cell injury that results in the release of intracellular contents into circulation. Commonly recognized causes of rhabdomyolysis can broadly be classified into traumatic and atraumatic etiologies. Atraumatic rhabdomyolysis has been linked to medications and recreational drugs such as alcohol, cocaine, and heroin. Chemotherapy-induced rhabdomyolysis has been reported with 5-azacytidine, cytarabine, pemetrexed, interferon-alpha with only case reports describing this condition with docetaxel [1-2]. Here we present a unique case of docetaxel-induced rhabdomyolysis, successfully managed with discontinuation of medication and conservative management.

## Case presentation

A 48-year-old woman presented to the ED for evaluation of severe bilateral lower leg pain and inability to walk. Her medical history is significant for diabetes mellitus and recently diagnosed lobular carcinoma of the left breast for which she had a left mastectomy with axillary dissection and prophylactic right mastectomy. Her tumor was grade III at diagnosis with negative margins and was 98% estrogen receptor (ER)-positive, 80% progesterone receptor (PR)-positive, 2+ for Her2Neu but FISH negative and Ki67 was 35%. CT scan and the bone scan did not show any distant disease. She was started on adjuvant docetaxel and cyclophosphamide chemotherapy with growth factor support given once every three weeks. She reported muscle pain in her bilateral lower extremities after cycle 2 and was started on non-steroidal anti-inflammatory drugs (NSAIDs) with mild improvement. Following cycle 3 the pain worsened and was sent to the ED for further evaluation. She denied any trauma to the legs. The patient denies smoking, drinking, or use of illicit drugs. Her home medications include insulin and losartan. 

On physical examination, the vitals were as follows: pulse rate of 128/min, respiratory rate of 18/min, temperature of 97.8 degree Farenheit, blood pressure 161/93 mmHg, and O2 saturation of 95% on room air. Her legs were tender on palpation bilaterally below the knee. There was no significant leg or joint swelling or erythema. Heart and lung examination were normal. 

**Figure 1:**
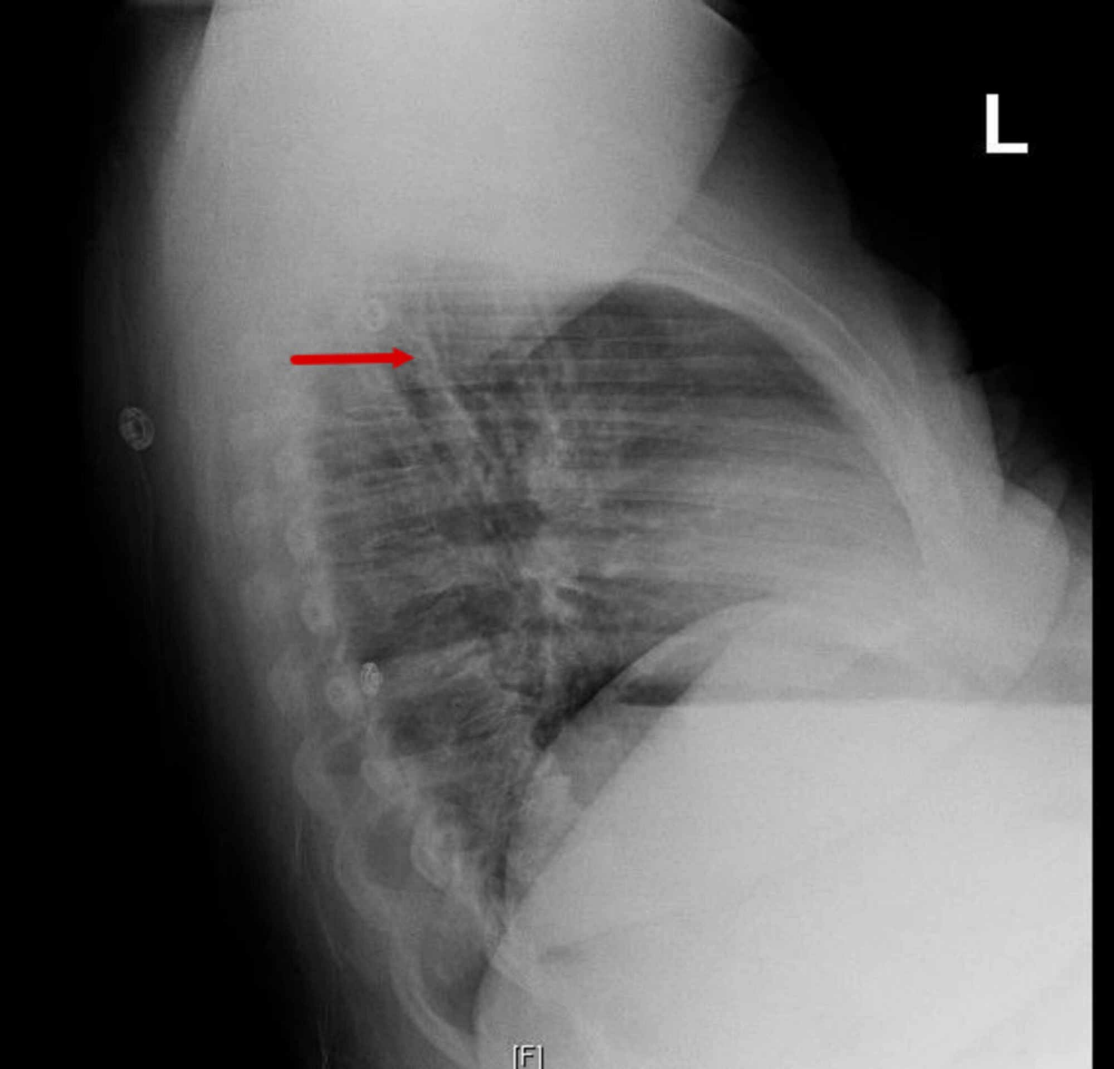
Lateral view of chest X-ray with findings suspicious for right upper lobe pneumonitis.

**Figure 2:**
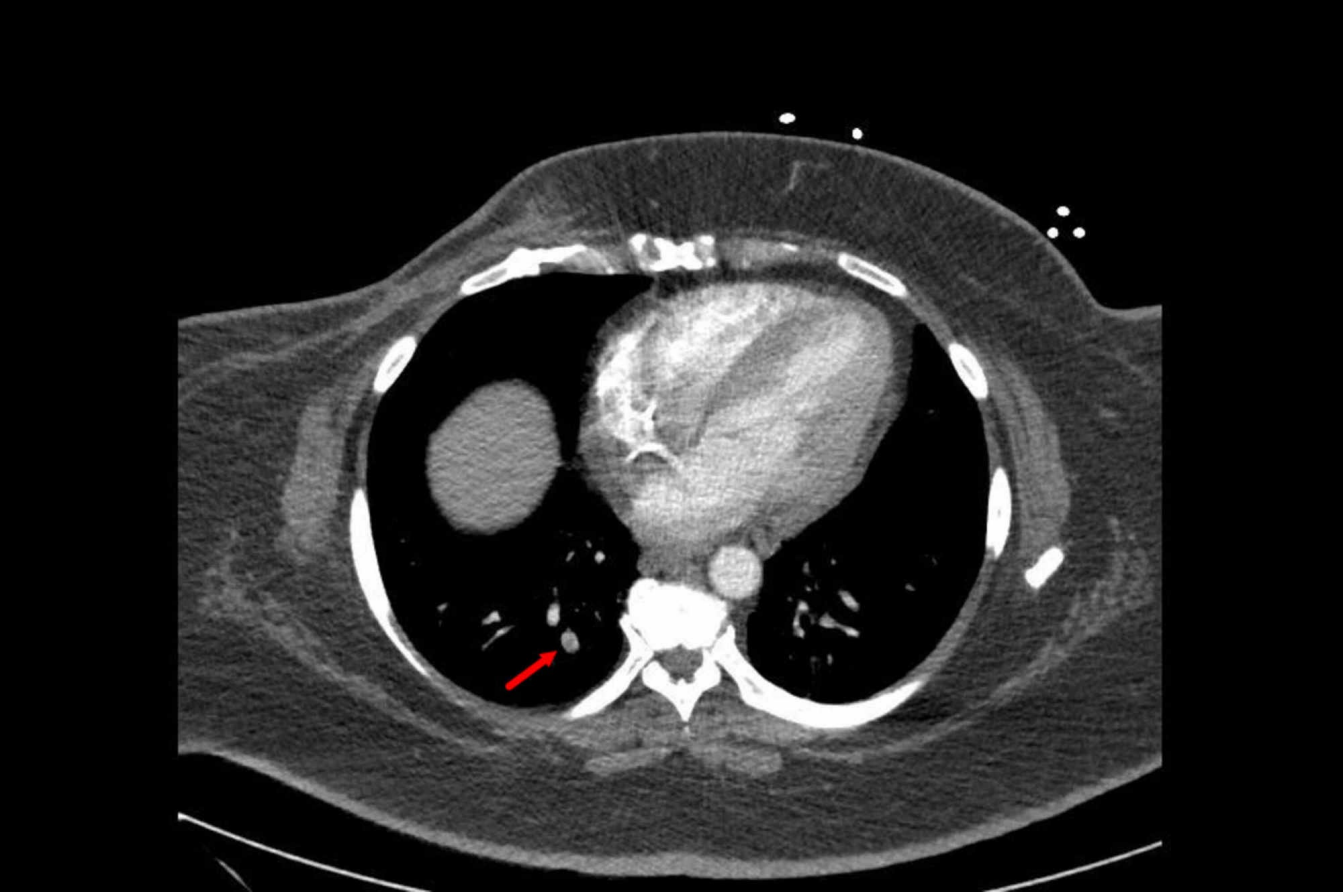
CT scan showing a small right lower lobe pulmonary embolus.

Initial blood work showed a normal kidney and liver function, along with normal electrolytes. Urine drug screening was negative. The creatine kinase (CK) levels were 1791 U/L with an myocardial band (MB) fraction of 0.6 U/L. Complete blood count (CBC) was unremarkable. Her thyroid stimulating hormone (TSH) and serum cortisol A.M. levels were normal and were 2.56 MIU/ML and 10 UG/DL respectively. Her chest X-ray had findings consistent with right upper lobe pneumonitis for which a CT chest was done that showed small right lower lobe pulmonary embolism with no pneumonia or pneumonitis (Figures 1-2).

 

**Figure 3:**
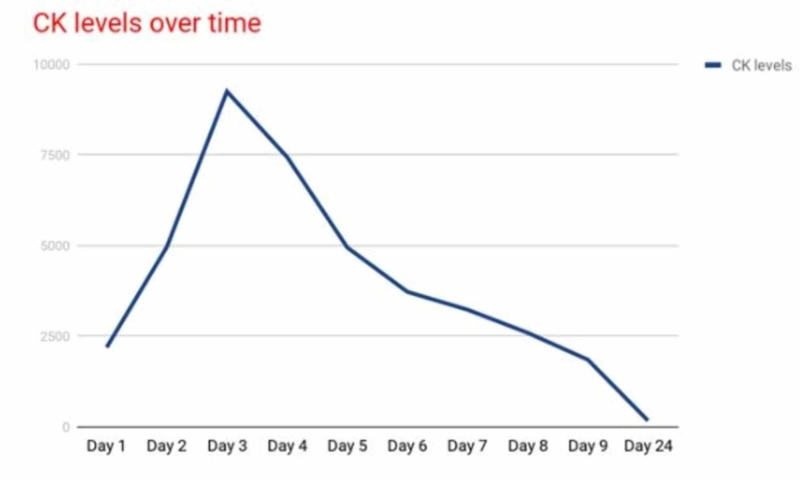
Trend of CK levels during and after the hospital stay. CK, creatinine kinase

The patient was started on enoxaparin and isotonic intravenous fluids. Throughout the stay, CK levels kept on rising and peaked at 9252 U/L (Figure 3).

She was treated with IV 0.9% normal saline infusion throughout her stay. She did not develop acute renal failure. She remained in the hospital until CK levels began to fall and the pain was more adequately controlled. Two weeks post-discharge, her CK levels normalized.

## Discussion

Rhabdomyolysis is a potentially life-threatening syndrome caused by injury to the skeletal muscle cells leading to leakage of its contents into the bloodstream and causing the characteristic triad of muscle pain, weakness, and dark urine [3]. It is caused by either traumatic insult in the form of exertion, crush injury, seizures, or nontraumatic insult in the form of alcohol, viruses, and drug toxicities [4]. Many physicians make a diagnosis of rhabdomyolysis when the CK levels are 5 or 10 times higher than normal levels as there is no fixed criterion to diagnose it [5].

**Table 1:** Most common causes of rhabdomyolysis.

Category	Commonly reported cause
Trauma	Crush syndrome
Exertion	Strenuous exercise, seizures, alcohol withdrawal
Muscle hypoxia	Limb compression during prolonged immobilization
Genetic defects	Disorders of glycolysis or glycogenolysis, disorders of lipid metabolism, mitochondrial disorders
Infections	Influenza A and B, Coxsackievirus, Epstein-Barr virus, primary human immunodeficiency virus, Legionella species, Streptococcus pyogenes, Staphylococcus aureus, Clostridium
Body-temperature changes	Heat stroke, malignant hyperthermia, malignant neuroleptic syndrome, hypothermia
Metabolic and electrolyte disorders	Hypokalemia, hypophosphatemia, hypocalcemia, nonketotic hyperosmotic conditions, diabetic ketoacidosis
Drugs and toxins	Statins, fibrates, alcohol, heroin, cocaine
Idiopathic	

Docetaxel is a chemotherapy drug and an analog of paclitaxel that belongs to the taxanes family. It is widely used in the treatment of multiple types of cancers which include breast, head and neck, stomach, prostate, and non-small-cell lung cancer [6]. The most common adverse effects of docetaxel noted from phase I & II clinical trials include myelosuppression, fluid retention, edema, and neuropathy [7]. There have been reports of some myositis cases in the literature, however, our extensive search showed no previous association of rhabdomyolysis with docetaxel. Perel-Winkler et al. described a 65-year-old ER- and PR-positive invasive ductal breast carcinoma who presented after the third infusion of docetaxel and cyclophosphamide with right thigh pain and found to have myositis with CK levels of 341 U/L and was subsequently managed by corticosteroids and discontinuation of docetaxel [8]. Ardavanis et al. also reported a case of a 57-year-old male on docetaxel and gemcitabine for advanced non-small cell lung cancer presented after the fourth cycle with bilateral symmetrical lower limb pain and swelling and was managed as acute myositis without rhabdomyolysis as the CK levels were not high [9]. Our patient’s CK levels peaked up to 9252 U/L which was consistent with rhabdomyolysis.

Docetaxel exhibits its cytotoxic effects by binding to microtubules with high affinity. This results in fewer microtubules available for mitotic activity and ultimately activates apoptosis of cells. It has been reported to cause myositis but not rhabdomyolysis. The mechanism of taxane-induced rhabdomyolysis is unclear. In a study conducted by Chaillou et al., on a murine model, docetaxel administered intravenously does not impair force production in skeletal muscles which led us to believe a different mechanism of myalgias [10]. One can hypothesize that docetaxel might share a similar mechanism as paclitaxel, which is another agent from the taxane family and has been reported to cause myositis and rhabdomyolysis in several cases [11].

Although the classical triad of rhabdomyolysis is not seen in our patient, we carefully reviewed and ruled out other differentials before considering docetaxel as the cause of rhabdomyolysis in our patient. Our patient had normal TSH and serum cortisol levels. Our patient showed a marked improvement with the discontinuation of docetaxel indicating it to be a culprit drug for the new-onset rhabdomyolysis. The treatment should be started early and the mainstay is aggressive IV fluids administration because of volume depletion from water sequestration in affected muscles. Our patient had rhabdomyolysis from a nontraumatic cause and we ruled out infections, metabolic derangements, thyroid abnormalities, and electrolyte disorders as other potential causes (Table 1). Acute kidney injury (AKI) which occurs in 4%-33% of patients is a grave complication of rhabdomyolysis, regardless of the cause and development of the same which is associated with poor prognosis [12-13]. Electrolyte abnormalities in rhabdomyolysis are due to the release of cellular contents and occur before AKI [3]. Our patient, however, did not develop any AKI or electrolyte derangements. She was treated with 0.9% normal saline alone and responded well.

## Conclusions

This is the first documented evidence of docetaxel-induced rhabdomyolysis. The management is similar to any other cause of rhabdomyolysis and includes discontinuation of the offending agent, aggressive IV fluids, and close monitoring of renal and electrolyte derangements. Clinicians need to be aware of this adverse effect of docetaxel treatment until more studies are available to clarify the causation and burden.
